# Low concentration DNA extraction and recovery using a silica solid phase

**DOI:** 10.1371/journal.pone.0176848

**Published:** 2017-05-05

**Authors:** Constantinos Katevatis, Andy Fan, Catherine M. Klapperich

**Affiliations:** 1 Division of Materials Science and Engineering, Boston University, Boston, Massachusetts, United States of America; 2 Department of Biomedical Engineering, Boston University, Boston, Massachusetts, United States of America; University of South Australia, AUSTRALIA

## Abstract

DNA extraction from clinical samples is commonly achieved with a silica solid phase extraction column in the presence of a chaotrope. Versions of these protocols have been adapted for point of care (POC) diagnostic devices in miniaturized platforms, but commercial kits require a high amount of input DNA. Thus, when the input clinical sample contains less than 1 μg of total DNA, the target-specific DNA recovery from most of these protocols is low without supplementing the sample with exogenous carrier DNA. In fact, many clinical samples used in the development of POC diagnostics often exhibit target DNA concentrations as low as 3 ng/mL. With the broader goal of improving the yield and efficiency of nucleic acid-based POC devices for dilute samples, we investigated both DNA adsorption and recovery from silica particles by using 1 pg– 1 μg of DNA with a set of adsorption and elution buffers ranging in pH and chaotropic presence. In terms of adsorption, we found that low pH and the presence of chaotropic guanidinium thiocyanate (GuSCN) enhanced DNA-silica adsorption. When eluting with a standard low-salt, high-pH buffer, > 70% of DNA was unrecoverable, except when DNA was initially adsorbed with 5 M GuSCN at pH 5.2. Unrecovered DNA was either not initially adsorbed or irreversibly bound on the silica surface. Recovery was improved when eluting with 95°C formamide and 1 M NaOH, which suggested that DNA-silica-chaotrope interactions are dominated by hydrophobic interactions and hydrogen bonding. While heated formamide and NaOH are non-ideal elution buffers for practical POC devices, the salient results are important for engineering a set of optimized reagents that could maximize nucleic acid recovery from a microfluidic DNA-silica-chaotrope system.

## Introduction

Nucleic acid (NA) molecular diagnostics are highly specific and sensitive assays that detect the presence or absence of target-specific DNA or RNA sequences from crude biological samples [1,2]. However, for fast and accurate results, these assays often require pure NA samples that are free of contaminants. As a result, commercial point of care (POC) kits typically employ a separate, upstream NA purification system in which DNA and RNA from crude samples are extracted and purified using solid phase extraction columns made from silica [3–5]. Although the adsorption strength and capacity of these silica columns have been well characterized in previous works, most studies were conducted within the confines of high NA loads, where the total input DNA exceeded 1 μg. Studies using smaller amounts of DNA in solution were performed using columns with < 200 μm^2^ cross-section [6–8], which increases the backpressure and potential for clogging, making them impractical for viscous biological samples like blood and sputum [9–12]. In the quest to improve the NA sensitivity and specificity of miniaturized POC devices for small-volume biological specimens, one must engineer a set of reagents and extraction protocols that would maximize both NA retention and elution from silica columns [2]. In clinical applications, obtaining high NA yield from such columns becomes challenging, since these samples often exhibit low NA copy numbers that are masked against a vast background of contaminants. For example, in urine (high salt), plasma (high protein), or cerebral spinal fluid, the concentration of NA may be as low as 3 ng/mL of sample [13].

The general workflow of molecular diagnostics can be summarized as the following: A biological sample, such as blood, urine, or cerebral spinal fluid, is treated with lysis buffer to liberate the NA from cells, bacteria, and/or virions. The DNA is then isolated from solution using a solid phase extraction column, retrieved using an elution buffer, and quantified via molecular tests for diagnosis. This method has two inherent loss mechanisms. First, DNA adsorption onto the column may be inefficient, and second, the purified DNA may not be efficiently eluted from the column. The mechanisms that govern the adsorption and elution of NA at low concentrations (in the absence of carrier molecules such as carrier DNA) are not well understood. Therefore, a better understanding of the surface chemistry of silica extraction columns is needed to engineer an NA extraction system that maximizes the final DNA yield and improves the diagnostic capability of POC NA extraction devices.

## Background

DNA extraction kits from silica-based solid phase columns often utilizes a chaotropic buffer that serves both as a protein denaturant and cofactor that promotes NA adsorption. A chaotrope is an ion that disrupts hydrogen bonding and disorders water molecules in an aqueous environment [14]. These ions are ranked within the Hofmeister series by their ability to enhance solubility of proteins. Thus, both hydration interactions and specific ion effects play a key role in dictating the order of the Hofmeister series [15,16]. In simple electrolyte solutions, when controlling for charge, this order manifests itself by the localization of the charge on a given ion [17]. Chaotropic ions exhibit increased charge delocalization that disrupts neighboring hydrogen bonding and directly leads to higher protein solubility in water. Consequently, chaotropic ions in water are usually associated with increased NA denaturation through base-pairing disruptions [18]. Previous work with DNA has shown that adding silica particles to a chaotropic solution will induce a pH-dependent DNA adsorption onto the particle surface [19].

The pH dependence of the DNA-silica-chaotrope adsorption results from the surface charge of silica and DNA [20]. Depending on the column fabrication method, the silica surface may contain a mixture of single silanols and/or geminal silanols with an isoelectric point around pH 1.5–3.6 [21]. Studies have shown the two effective acid dissociation constants (pK_a_) of silica silanols are pH 4.5 and 8.5 [22,23]. In the context of NA extraction from biological samples at a physiological pH of 7.0–7.4, the vast majority of the exposed silica surfaces should theoretically be covered with negative charges. Moreover, since DNA has an isoelectric point near pH 5, it too is predominantly negatively-charged at physiological pH. Hence, it is very difficult to explain the apparent silica–DNA affinity within NA extraction columns using electrostatics arguments alone [15,16,24]. In terms of microfluidic medical diagnostics research, one must attempt to understand the fundamental surface chemistry by quantifying the interplay among DNA, silica, and chaotropic molecules as a function of pH to fully optimize the NA extraction yield from complicated heterogeneous biological samples [17].

Previous work has examined the DNA-silica-chaotrope interactions at concentrations of DNA in excess of 1 μg/mL [19,25,26]. In these experiments, the high input DNA concentrations often saturated the silica surface [19,26]. Thus, it is very difficult to extrapolate from these results to NA extraction protocols for common clinical samples that do not saturate the silica surface: for example, urine, plasma, and cerebrospinal fluid have DNA concentrations of 40–200 ng/mL, 17 ng/mL, and 3 ng/mL DNA respectively [13,27,28]. Thus, it is important to understand the DNA-silica-chaotrope interaction when the DNA in solution is the limiting reagent. While using miniaturized silica columns with small pores could lead to more efficient adsorption and elution for dilute NA samples, these columns can be susceptible to clogging or surface passivation by other biomolecules such as proteins, lipids and carbohydrates [12,29].

Here, we aim to characterize the DNA-silica-chaotrope interactions, at clinically-relevant DNA concentrations, as a function of pH. The pH values of 3, 5.2, and 8 were chosen on the basis of the pK_a_ values of the silica surface groups, the depurination of DNA (below pH 2), and the dissolution of silica (above pH 8) [30]. DNA adsorption and elution from the silica surface was quantified under conditions that mimic those commonly used in commercial kits and integrated POC diagnostic devices that isolate NAs from human samples [31–33]. However, we incubated the DNA and silica particles significantly longer than most test protocols call for to make sure that equilibrium was reached. To further focus the scope of our investigation, we only considered commercial silica particles made for biological assays, and λ-phage DNA as the input NA. Finally, we restricted the chaotropes used to the guanidinium (CH_6_N_3_ or Gu) and thiocyanate (SCN) ions, commonly used in commercial NA extraction kits [4,34].

## Materials and methods

### Materials

All experiments were conducted using Davisil 643 amorphous silica particles (Sigma-Aldrich, St. Louis, MO, USA) and genomic λ-DNA (New England BioLabs, Beverley, MA, USA) with molecular mass of 52.3 ag per copy according to the manufacturer. Stock buffer solutions for pH 3 and 5.2 were made by titrating 1 M sodium formate and 1 M sodium acetate with their conjugate acids, respectively. Stock 1 M tri(hydroxymethyl)aminomethane (Tris) buffer was adjusted to pH 8 with HCl. All chemicals were purchased from Sigma-Aldrich. Solutions used to adsorb DNA onto silica were made from desired stock buffer solutions to final concentrations of 225 mM buffering ion and 5 M GuSCN. Similar solutions were also made without GuSCN and used as a no-chaotrope control. The six adsorption solutions that were tested are described in Table 1. Adsorption solution A and D had 225 mM sodium formate (pH 3), B and E had 225 mM sodium acetate (pH 5.2), and C and F had 225 mM Tris-Cl (pH 8), while D, E, and F also contained 5 M GuSCN.

**Table 1:** Adsorption solutions tested in experiment. Adsorption of DNA on silica was tested in six different adsorption solutions. Solutions at pH 3, 5.2, and 8 were buffered with 225 mM sodium formate, sodium acetate, and Tris-Cl respectively.

Adsorption solution	Chaotrope	pH	Buffering agent
A	0 M GuSCN	3	225 mM HCOONa
B		5.2	225 mM CH_3_COONa
C		8	225 mM Tris-Cl
D	5 M GuSCN	3	225 mM HCOONa
E		5.2	225 mM CH_3_COONa
F		8	225 mM Tris-Cl

### DNA-silica system

**Fig 1:**
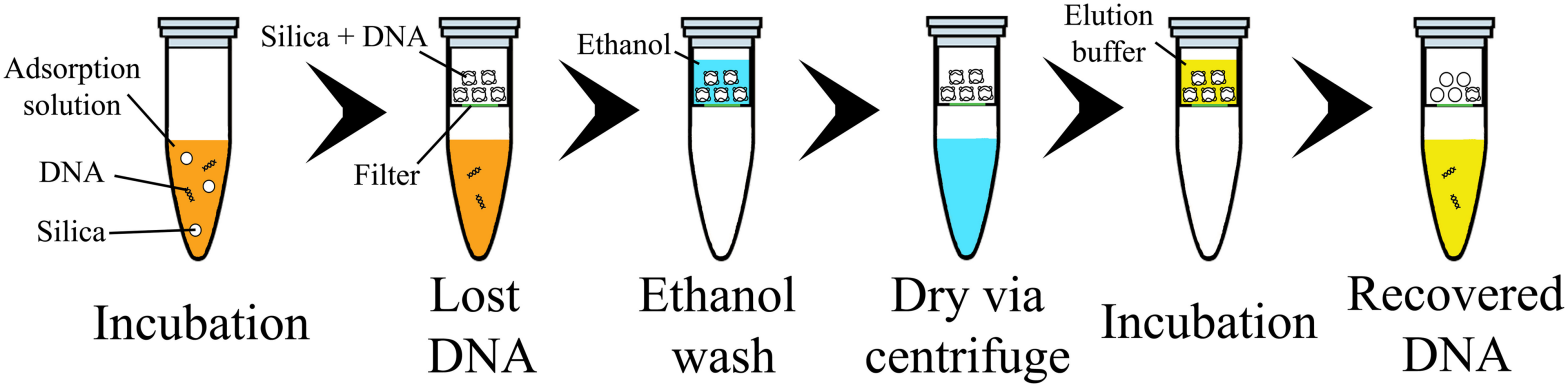
Schematic of general protocol. Silica, adsorption solution, and DNA were combined in a microcentrifuge tube. This solution was incubated for 1 hour in an agitated state to promote mixing. The silica particles and any DNA adsorbed onto them were separated from solution using a filter. The supernatant was analyzed to determine the ‘Lost DNA’ (DNA amount not adsorbed after incubation). The particles were subsequently washed with 70% ethanol and dried. The elution buffer was then added and incubated for 5 min before it was also collected to determine the ‘Recovered DNA’ (DNA amount eluted from the silica surface.

Using NA extraction and elution protocols from standard Qiagen extraction kits and previously published POC diagnostic devices as templates, the overall experimental procedures are shown in Fig 1. In brief, silica particles were first added to 1.7 mL microcentrifuge tubes by dispensing 10 μL of a colloidal solution made from 50 mg Davisil 643 silica particles suspended in 220 μL ethanol. The 10 μL aliquot was allowed to dry in an ambient environment for 1 hour. This step resulted in tubes containing 2.2 ± 0.2 mg (n = 18) of silica particles possessing a theoretical total surface area of 0.66 ± 0.06 m^2^ and a dry-packed column volume (CV) of 3.6 ± 0.3 μL (n = 24). Verification of the latter parameter was performed by measuring the effective height of the silica column when the same 10 μL aliquot was packed and dried in a cylindrical glass capillary with an inner diameter of 580 μm. Once the ethanol evaporated completely, 390 μL of the adsorption solution and 10 μL of λ-DNA solution were added to the silica particles. The net mass of input DNA ranged from 1 pg to 1 μg, and corresponded to DNA concentrations between 2.5 pg/mL to 2.5 μg/mL (4.8·10^4^ copies/mL to 4.8·10^10^ copies/mL). The contents of each tube were mixed by inversion, placed on a tube rotator, and incubated at room temperature for 1 hour to allow DNA to adsorb onto the silica surface. The silica particles were separated from the liquid phase using Millipore Ultrafree MC 0.22 μm centrifugal filters (Billerica, MA, USA) spun for 2 min at 12,100 x g. The aqueous filtrate was collected to quantify the DNA amount not adsorbed during the adsorption step: we define this as ‘Lost DNA’ since it was not captured by the column. The silica particles were then washed with 400 μL 70% ethanol and spun again to dry the particles further. Qiagen buffer EB (10 mM Tris-Cl at pH 8.5, Qiagen, Valencia, CA, USA) was used as the elution buffer for all adsorption conditions. Buffer EB (10 mM Tris-Cl, pH 8.5) is a standard buffer in solid phase extraction kits due to its high pH and low salt properties [4]. For the elution step, 400 μL buffer EB was added to the silica particles, incubated for 5 min at ambient conditions, and then spun down. The resulting filtrate was collected to quantify the DNA eluted from the silica particles: we define this as ‘Recovered DNA’ this would be the purified DNA used for downstream quantification methods if this protocol was used in a diagnostic device. Since the elution volume is more than 400 times the CV of the silica particles, we expect the DNA elution peak to be located within the first eluate. As a negative control, adsorption solution containing λ-DNA was also passed though the Millipore filters (without silica), collected, and quantified to assess how much DNA might be lost by adsorption to the filter. The range of adsorption solutions tested are listed in Table 1 and consisted of pH 3, 5.2, and 8, each with and without GuSCN. No-DNA control reactions were also tested. GuSCN was chosen as the chaotrope due to its common use in cell lysis protocols [3,35].

### Elution curves

To understand the DNA-silica-chaotrope interaction, elution curves were generated for different conditions. For selected DNA adsorption solutions, the post-adsorption silica particles were subsequently washed, dried, and eluted with 4 serial volumes of a selected elution buffer. Four elution buffers were chosen for their ability to disrupt specific intermolecular interactions: 1) 0.5 M Tris-Cl (pH 8.5) to disrupt electrostatic interactions, 2) 95°C formamide to disrupt hydrogen bonding, 3) 1 M NaOH to release DNA by dissolving the silica surface, and 4) buffer EB to deprotonate the phosphate and silanol surface groups on DNA and silica respectively due to its pH buffering capability. Nuclease-free water was also used to elute DNA from silica as a control.

### DNA purification

Since the polymerase chain reaction (PCR) was used to quantify the DNA amounts lost and recovered, an additional ethanol precipitation step was applied to all filtrates to remove PCR-inhibiting salts [36]. To elaborate, filtrates were placed in 1.7 mL microcentrifuge tubes containing 5 μL of 20 mg/mL glycogen (Thermo Scientific, Waltham, MA, USA), 30 μL of 5 M NaCl, and 65 μL of buffer EB to a total volume of 500 μL. The solutions were vortexed, allowed to sit for 2 min at room temperature, and then 1.1 mL of ethanol was added and mixed to precipitate the DNA. After another 2 min of room-temperature incubation, solutions were spun at 17,900 x g for 15 min at 22°C to generate glycogen-DNA pellets. Upon discarding the supernatant, the pellets were washed twice with 500 mL 70% ethanol. After discarding the supernatants, the glycogen-DNA pellets were allowed to dry in ambient conditions for 15 min. Finally, the pellets were resuspended in buffer EB. For DNA filtrate samples containing formamide, the protocol was changed to account for the higher polarity of formamide compared to water. The 1.1 mL of ethanol during precipitation was replaced with 833 μL of tert-butanol. As an additional control, ethanol precipitation from solutions with known amounts of input DNA was carried out as positive controls to ensure efficacy of precipitation process.

### qPCR

**Fig 2:**
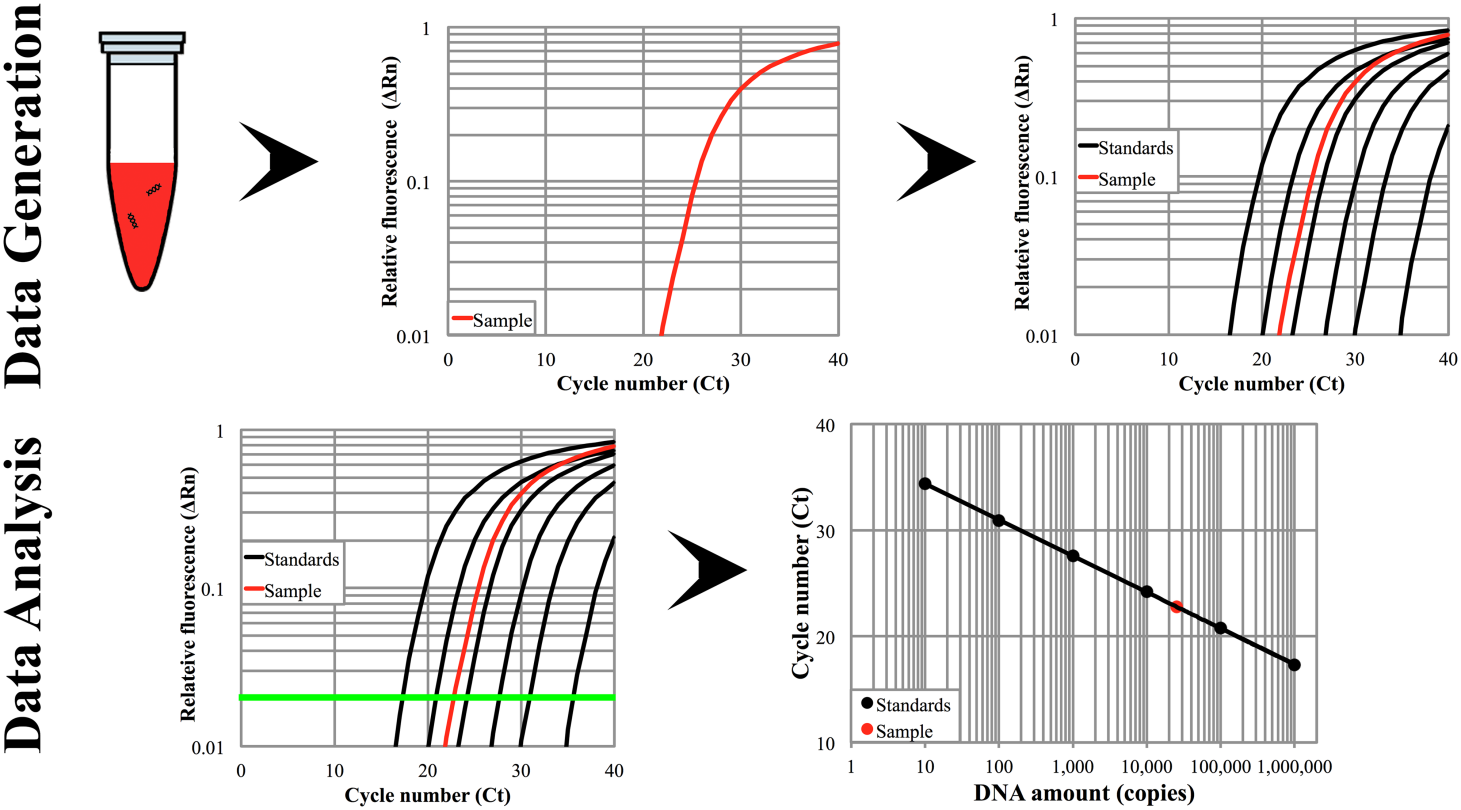
Schematic of qPCR data analysis. Relative fluorescence (ΔRn) was recorded at each PCR cycle for samples and standards of known DNA amounts. A ΔRn threshold value was chosen (green line) in the linear region to determine a Ct value. The Ct value is plotted vs. the amount of input DNA and fit with a regression line. This line was used to back-calculate the initial amount of DNA in an unknown sample by plotting sample on the line with its Ct value.

DNA loss and recoveries from each experiment were ascertained via quantitative polymerase chain reaction (qPCR). Samples were amplified using SureStart Taq polymerase (Agilent, Santa Clara, CA, USA) using an Applied Biosystems 7500 thermocycler. Custom primers and Taqman probe sequences were designed for specific sequences of λ-DNA. The forward primer was (5'- GTG GAA TGA ACA ATG GAA GTC AAC AA -3'), the reverse primer was (5'- GGC AGA GTC ATA AAG CAC CTC ATT A -3') (Integrated DNA technologies), and the Taqman probe was (5'- AGG TGC TAC GGC GGC AGA GT -3') tagged with 6-FAM at the 5’-end and a MGB-NFQ quencher at the 3’-end (Applied Biosystems, Waltham, MA, USA). The resulting amplicon was 177 base pairs long. Each 25 μL reaction contained 5 μL of the purified DNA filtrates. Samples were placed in a 96-well plate (Applied Biosystems, Waltham, MA, USA), initially heated to 95°C to activate the polymerase and then cycled 40 times through 30 sec of 95°C for DNA denaturing, 5 sec of 65°C for primer annealing, and 30 sec of 72°C primer extension. qPCR generates curves of the relative fluorescence (ΔRn) after each temperature cycle. The amount of starting DNA is calculated based on the cycle at which the fluorescence in a given well reached a threshold value as shown in Fig 2.

## Results and discussion

### Strongest DNA adsorption occurs at low pH with GuSCN

**Fig 3:**
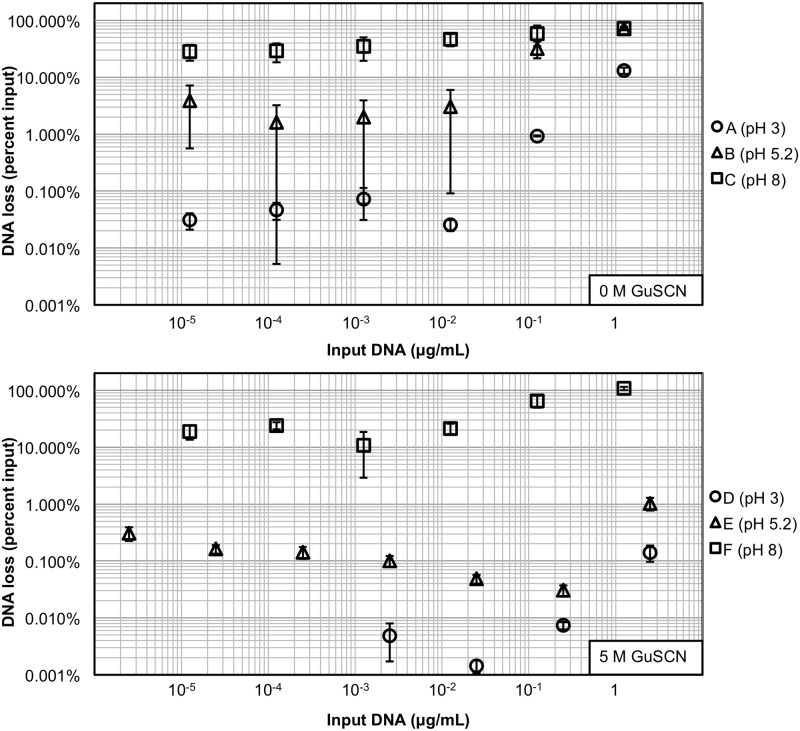
Lost DNA following incubation with silica. Plots show amount of DNA not adsorbed to silica after 1 hour of incubation for each adsorption solution. Solutions tested were pH 3 (circles), 5.2 (triangles), and 8(squares), and solutions D, E, and F also contained 5 M GuSCN. Note that DNA loss for 2.5, 25, and 250 pg/mL are not shown for adsorption solution D, since the signal from those values for unadsorbed DNA fell below that of the no DNA control. (n = 3).

To analyze the adsorption efficiency of DNA on silica, we explored the amount of DNA lost (never bound to the solid surface during the first incubation step). We began by examining the DNA loss mechanisms. In Fig 3, we observed that regardless of the presence or absence of GuSCN during the DNA adsorption process, the highest DNA loss occurred at pH 8. Since pH 8 is significantly above the first silanol pK_a_ (4.5) and near the second pK_a_ (8.5) [20], the data supports the theory in which an overall negatively-charged silica surface is electrostatically repelling the negative-charged DNA phosphate backbone. Moreover, the data also supports previous studies, in which silica dissolution at pH 8–10 inhibits DNA adsorption [30]. In essence, the pH 8 data reinforces the common observation that commercial NA extraction kits perform poorly at high pH [19,34].

Next, we observed an overall enhanced DNA adsorption at pH 3 and 5.2. The addition of GuSCN increased adsorption by an order of magnitude at these pH values. For input DNA less than 25 ng/mL at pH 3, the amount of DNA loss during initial adsorption was less than 0.1%. At higher input DNA concentrations, the percent loss also increased, presumably due to the onset of surface saturation by the excess DNA molecules. Similar results are seen at pH 5.2, albeit with the initial loss of 1.6–3.8%. This supports the theory that surface charges inhibit DNA-silica adsorption. Since isoelectric points of DNA and silica are 5 and 1.5–3.6 respectively, the decrease in the level of negative-negative charge repulsions would presumably make DNA-silica interactions more favorable in an acidic environment. The data supports this expectation, since DNA loss was the lowest for pH 3, increased for pH 5.2, and reached a peak at pH 8, controlling for the presence of GuSCN. The error bars for DNA adsorbed at pH 5.2 without GuSCN are the largest in the plot. The standard deviations ranged 0.7–10.4%, which are less than the pH 8 standard deviations that ranged 4.8–23.2% for the same input concentrations. Adsorption at pH 3 was stronger and resulted in smaller standard deviations (0.01–1.7%), suggesting that adsorption at pH 5.2 is of strength between that at pH 3 and pH 8.

### Optimal DNA recovery when adsorbed with 5 M GuSCN at pH 5.2

**Fig 4:**
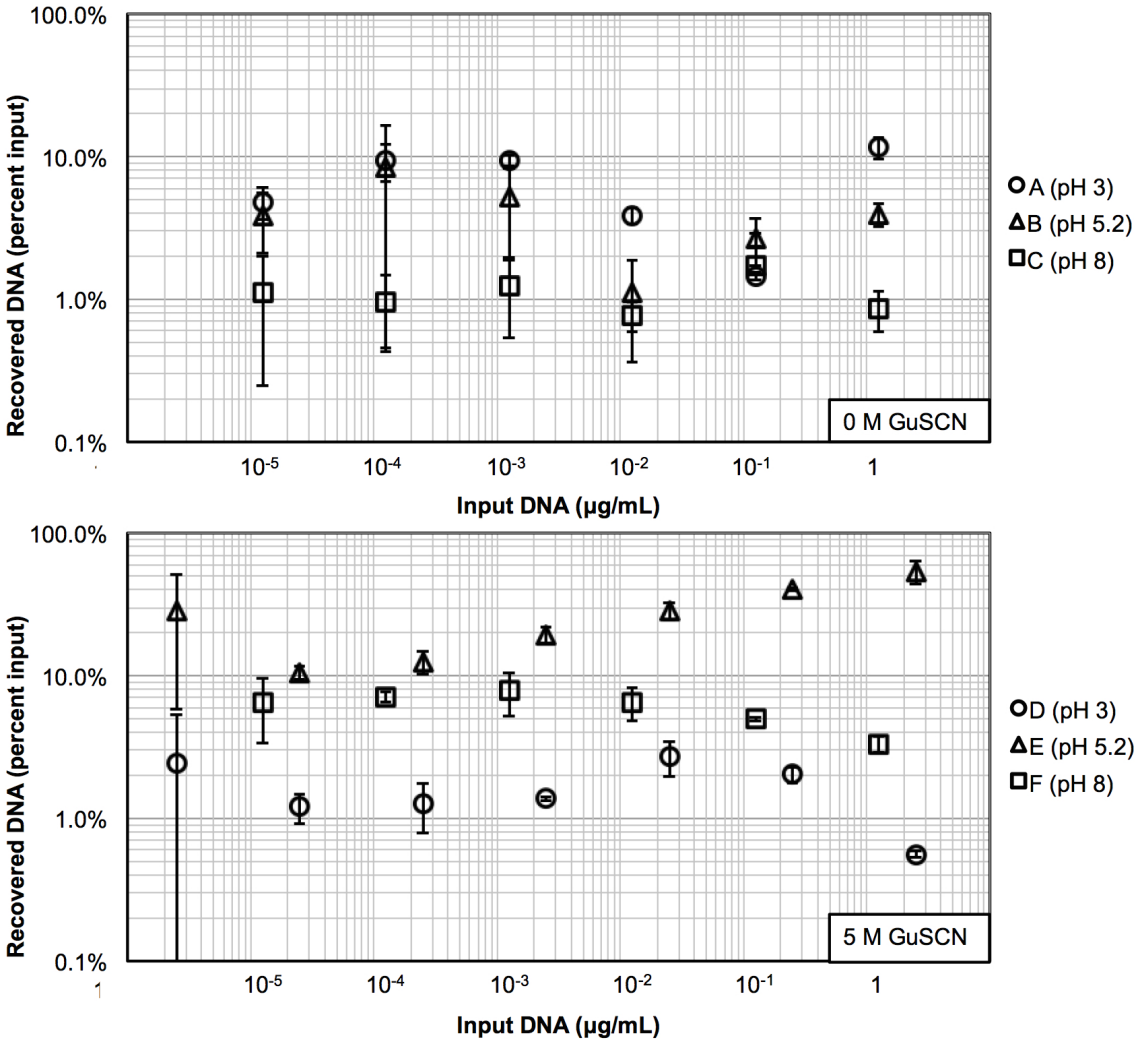
Recovered DNA with buffer EB elution from silica. The plots show DNA recovered from the silica surface after DNA incubation with each adsorption solution. Solutions tested at pH 3, 5.2, and 8 are labeled as circles, triangles, and squares respectively, and solutions D, E, and F also contained 5 M GuSCN. All samples were eluted with buffer EB (10 mM Tris-Cl pH 8.5). (n = 3).

Here, we analyzed the elution efficiency through the amount of DNA recovered (liberated from the solid phase with the addition of elution buffers). In Fig 4, all samples were eluted with buffer EB (10 mM Tris-Cl, pH 8.5), which is the gold standard for DNA-silica extraction columns. Across all DNA concentrations, the DNA recovery ranged between 0.8–11.54% for adsorption solutions A, B, and C (no chaotrope). These results suggest that eliminating GuSCN during adsorption would render at least 71% of DNA to be deemed unrecoverable during the entire extraction process. Such losses make these conditions unfavorable for clinical applications.

On the other hand, the addition of GuSCN appeared to improve the levels of DNA adsorption and recovery. When the input DNA concentrations was less than ≤ 2.5 ng/mL, DNA recovery from adsorption solutions D, E, and F (with GuSCN) exhibited similar behavior of adsorption solution groups A, B, and C (without GuSCN). In these cases, the recoveries ranged from 1.2–28.3%. For input concentrations ≥ 2.5 ng/mL, the DNA recovery did not improve when DNA was adsorbed using solutions D and F. Finally, DNA recovery following adsorption with solution E (pH 5.2), increased to 40.3% and 53.5% for 250 ng/mL and 2.5 μg/mL input DNA respectively. Since results from Fig 3 showed that the strongest adsorption occurred at pH 3, we suspect the weaker adsorption at pH 5.2 (Fig 3) facilitated the corresponding observed improvement in elution efficiency in Fig 4.

### The DNA-silica-chaotrope interaction at low pH is dominated by hydrophobic forces

In our series of experiments, the largest percentage of input DNA recovered was 69.3%, which is far from ideal for clinical applications. In an effort to improve this yield, we studied the effectiveness of different elution buffers. We generated elution curves using 1M NaOH, 95°C formamide (hot formamide), 0.5 M Tris-Cl, buffer EB, and water, following a 1 μg DNA adsorption step using solution D. It was chosen since it generated the strongest DNA-silica-chaotrope complex prior to the elution process, this allowed us to deconstructively interrogate specific molecular interactions within the complex by using reagents that specifically disrupted hydrogen bonding, electrostatic, or van der Waals forces. In Melzak *et*. *al*, the prevailing theory of chaotrope-mediated DNA adsorption on silica states: At low pH, where adsorption is strongest, DNA is driven onto the surface through dehydration and the formation of hydrogen bonds [19]. This theory was based on how globular proteins adsorbed to polystyrene [37]. For this to be true, we hypothesized that 1 M NaOH (pH 13) would recover the most DNA through high-pH silica dissolution. Likewise, elutions with 95°C formamide (hot formamide) would liberate DNA on the basis of hydrogen bond disruptions. If the combination of hot formamide and 1 M NaOH (pH 13) resulted in maximized DNA recovery, it would support the hypothesis that hydrogen bonding and hydrophobic interactions dominate the DNA adsorption process. Finally, water, buffer EB, and 0.5 M Tris-Cl would target possible ionic interactions within the system.

**Fig 5:**
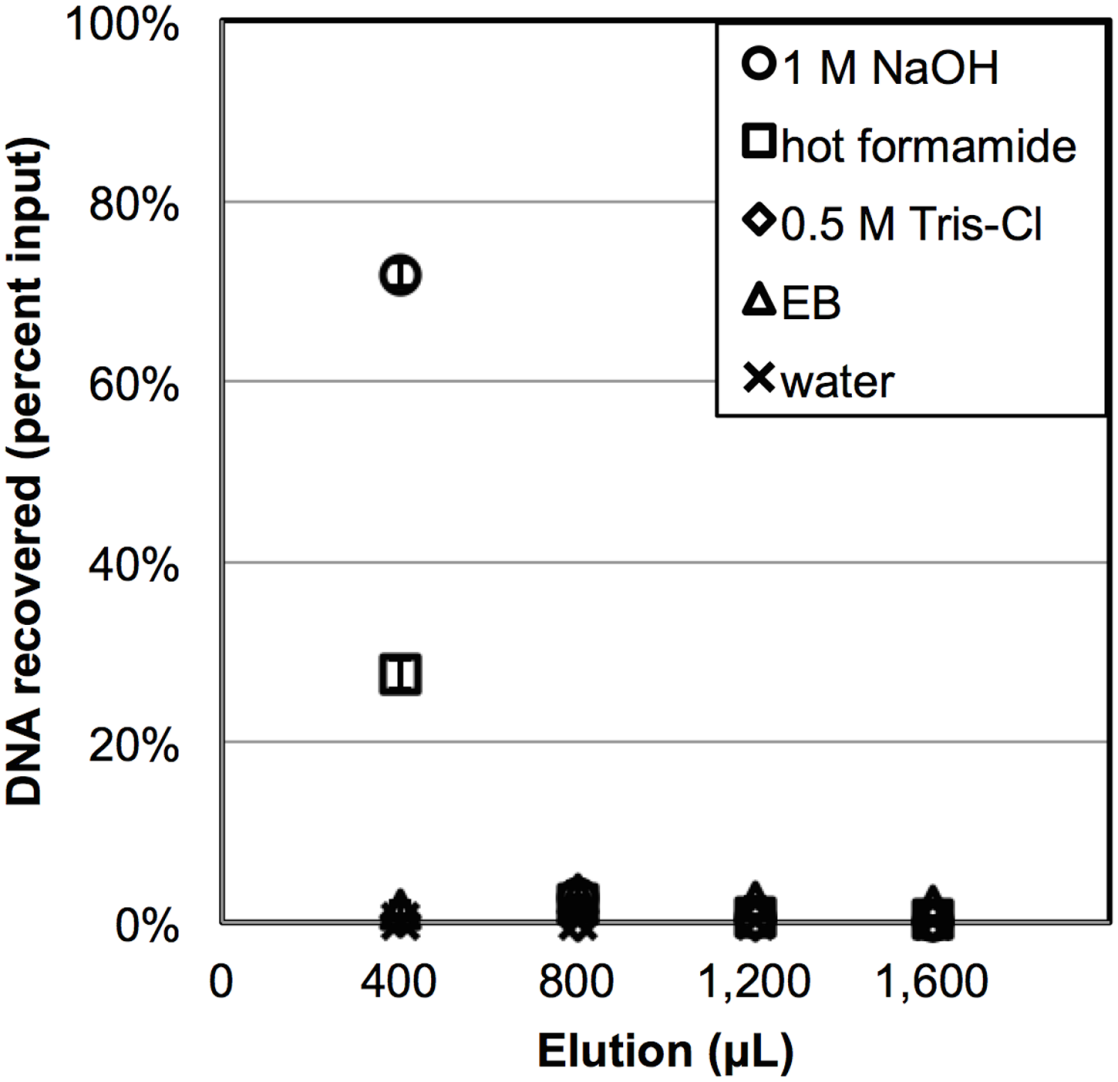
Elution of DNA from silica using varying elution buffers. Amount of recovered DNA using five different elution buffers. Samples were eluted after 1 μg DNA was incubated with silica in adsorption solution D (5 M GUSCN pH 3). (n = 3).

The elution curve data is shown in Fig 5. As hypothesized, the 1 M NaOH and 95°C formamide elutions resulted in the largest DNA recoveries with 71.9% and 27.5% respectively in the initial elution volume. Recoveries dropped to < 3% following the first elution fraction. For all other elution buffers, the DNA recovery ranged between 0.04–2.63%. Next, when analysis of variance (ANOVA) was used to compare the elution buffer efficacy for the first elution fractions. At least one of the elution buffers exhibit a statistically-different DNA recovery percentage among the group of five buffers [F_(4,10)_ = 2649, P < 10^−17^]. Furthermore, an *a posteriori* Tukey test showed that DNA recovery using 1 M NaOH and 95°C formamide were significantly different than recoveries using buffer EB, water, and 50 mM Tris-Cl (pH 8.5) at α = 0.01. In addition, there was a significant difference in DNA recovery using 1 M NaOH versus 95° formamide at α = 0.01.

## Discussion

The DNA loss data suggests that DNA adsorption on silica is mainly dependent on pH. DNA has an inherently greater affinity for the silica surface in acidic environments. However, the addition of the chaotrope (GuSCN) increased the surface affinity for DNA at pH 3 and 5.2. We expected this result because the isoelectric points of DNA and silica are 5 and 1.5–3.6 respectively. At pH 3, we did not expect electrostatics to interfere with the chaotropic mechanism that increased DNA adsorption, resulting in the highest surface affinity. Both the DNA and the silica surface become more negative at pH 5.2, and the corresponding increase in electrostatic repulsion resulted in more DNA loss. At pH 8, the loss was maximized. It has been theorized that chaotropes increase DNA-silica affinity by dehydrating the surface and promoting hydrogen bonding between the DNA and silica surface [19]. Our DNA loss results were consistent with previous studies using higher concentrations of DNA [15,19].

Focusing on events downstream of the DNA adsorption process, our elution results suggest that an increase in DNA adsorption capacity at low pH does not necessarily leads to a higher DNA recovery rate. Instead, the best DNA recovery results were obtained by taking advantage of a weaker DNA-silica-chaotrope adsorption complex facilitated at the intermediate pH of 5.2. Buffer EB, the gold-standard, was not able to disrupt the DNA-silica-chaotrope complex created at pH 3 since DNA recovery did not exceed 10.1%. Conversely, when the DNA-silica-chaotrope complex was formed at pH 8, weak DNA adsorption events resulted in massive 48.8% DNA loss prior to the EB elution steps. Thus, a DNA-silica-chaotrope adsorption event at pH 5.2 represents an optimum situation where: (A) The initial DNA loss due to a weaker solid-phase complex is offset by (B) An ease in complex dissociation with buffer EB at pH 8.5, thus resulting in a multi-factor local maximum in total DNA recovery.

Diving deeper into Fig 5, a maximum DNA recovery with 1 M NaOH suggests that hydrophobic forces dominate DNA-silica-chaotrope association events in a pH 3 environment. In arriving to this conclusion, we first employed buffer EB and 0.5 M Tris-Cl (pH 8.5) in an attempt to induce DNA repulsion and remove DNA from the silica by deprotonating both the DNA phosphate groups and surface silanol groups. Unfortunately, > 98% of the captured DNA remained on the surface, and only 2% of the bound DNA was recovered. Next, a 95°C formamide solution was used disrupted the hydrogen bonding between the DNA and silica surface, resulting in a higher recovery of 27.5%. Finally, eluting with 1 M NaOH resulted in a 71.9% recovery. Since NaOH completely destroys the DNA-silica-chaotrope complex via silica surface dissolution, the high level of NaOH-dependent DNA liberation suggests that hydrophobic interactions plays a larger role than hydrogen bonding or ionic interactions in the processes of adsorption complex formation at pH 3. We expected the lack of ionic interactions was due to the fact that phosphate and silanol surface groups are protonated and charge-neutralized at pH 3. Thus, if the destruction of all possible hydrogen bonds with hot formamide only amounted to liberating a small portion of the entire pool of adsorbed DNA molecules, then hydrophobic interactions are the likely dominant force that holds the DNA-silica-chaotrope complex together.

The experiments were conducted such that DNA, rather than the available surface, was the limiting factor for adsorption. Existing literature has shown that the adsorption capacity for DNA per unit surface of silica is 240–800 μg/m^2^ in the presence of a chaotrope, depending on the pH of the adsorption solution [6,19,26]. Our results support the model in which the adsorption capacity increases with decreasing pH. Due to experimental design constraints, the maximum theoretical DNA adsorption we could achieve for 1 μg total DNA was 1.52 μg/m^2^. In this regime when using GuSCN, no appreciable DNA was adsorbed at pH 8 and no appreciable DNA will be eluted when adsorbed at pH 3. This is in contrast to other experimental designs in literature that have demonstrated recovery of significant amounts of DNA after adsorbing at pH 4 or 8, with the caveat that the amount of unrecoverable DNA within the system was not addressed. To elaborate, these studies were conducted using large concentrations of input DNA, such that the net unrecoverable amount of DNA was negligible compared to the net recovered [6,25,26]. These differences become vital when one is trying to use silica columns to bind and release minute amounts of total DNA from NA-dilute clinical samples. Of course, most commercial kits address this problem by adding exogenous DNA to artificially increase the overall DNA load. However, this approach is not ideal in POC applications, since adding reagents increases cost, complexity, and volume to devices that need to be as inexpensive, simple, and small as possible.

Our results suggest that the standard method for eluting DNA using buffer EB from silica following chaotrope-mediated adsorption may not be ideal for low concentration of DNA. Only two conditions allowed for the recovery of more than 30% of input DNA with buffer EB. Based on these results, DNA recovery depends on 1) the concentration of DNA in the adsorption solution, 2) the adsorption solution pH, 3) the presence of a chaotrope, and 4) the elution buffer. It also depends on the ratio of input DNA to the available surface area of silica when comparing to previous results. This however was controlled for in these experiments. Thus, designing a device to use this technology requires not only an understanding of the DNA-silica-chaotrope interaction, but the dynamic range of characteristics associated with clinical samples and how sample preparation steps affect these characteristics. Our data suggests that we can increase the range of initial sample conditions for which this technology can be implemented.

## Conclusions

We developed a protocol to assess the adsorption and elution of DNA from silica particles while emulating POC DNA extraction conditions for samples containing ≤ 1 μg DNA. Controlling for presence of a chaotrope, DNA adsorption increased with increasing acidity of the aqueous adsorption solution. Adsorption was further enhanced by adding 5 M GuSCN to the adsorption solution. Elutions exceeded 30% DNA recovery only when DNA was adsorbed at pH 5.2 with 5 M GuSCN for inputs of ≥ 100 ng net DNA. Adsorption at pH 3 resulted in DNA-silica-chaotrope complex that was too strong for buffer EB to disrupt effectively, while adsorption at pH 8 was not strong enough for DNA to adsorb sufficiently. Due to the poor recover of DNA with buffer EB at pH 3, we attempted to disrupt DNA-silica-chaotrope complex by varying the eluting solution. Hot formamide and 1 M NaOH resulted in increased recovery of 27.5% and 71.9% respectively, leading to the conclusion that the interaction was dominated by hydrophobic interactions and hydrogen bonding. However, neither of these buffers is practical for POC use. The optimal adsorption condition for POC use studied here was achieved using pH 3 with 5 M GuSCN resulting in up to 99.998% DNA adsorbed, while the optimal DNA recovery with buffer EB was 53.5% DNA using pH 5.2 with 5 M GuSCN. As our knowledge of these interactions improve, so will the range of clinical applications for this technology.
